# Anti-Inflammatory and Gut Microbiota Modulatory Effect of *Lactobacillus rhamnosus* Strain LDTM 7511 in a Dextran Sulfate Sodium-Induced Colitis Murine Model

**DOI:** 10.3390/microorganisms8060845

**Published:** 2020-06-04

**Authors:** Soyoung Yeo, Hyunjoon Park, Eunsol Seo, Jihee Kim, Byoung Kook Kim, In Suk Choi, Chul Sung Huh

**Affiliations:** 1 WCU Biomodulation Major, Department of Agricultural Biotechnology, College of Agriculture and Life Sciences, Seoul National University, Seoul 08826, Korea; ysso1215@gmail.com (S.Y.); eunsol1013@snu.ac.kr (E.S.); jiheek@ckdbio.com (J.K.); 2 Research Institute of Eco-Friendly Livestock Science, Institute of Green-Bio Science and Technology, Seoul National University, Pyeongchang 25354, Korea; hyunjoons@snu.ac.kr; 3 Chong Kun Dang Bio Research Institute, Chong Kun Dang Bio Research Institute, Ansan 15604, Korea; bkkim@ckdbio.com (B.K.K.); inschoi6@ckdbio.com (I.S.C.); 4 Graduate School of International Agricultural Technology, Seoul National University, Pyeongchang 25354, Korea

**Keywords:** *Lactobacillus rhamnosus*, probiotics, dextran sulfate sodium, intestinal inflammation, gut microbiota, dysbiosis

## Abstract

Inflammatory bowel disease (IBD) is a group of conditions involving chronic relapsing-remitting inflammation of the gastrointestinal tract with an unknown etiology. Although the cause–effect relationship between gut microbiota and IBD has not been clearly established, emerging evidence from experimental models supports the idea that gut microbes play a fundamental role in the pathogenesis of IBD. As microbiome-based therapeutics for IBD, the beneficial effects of probiotics have been found in animal colitis models and IBD patients. In this study, based on the dextran sulfate sodium (DSS)-induced colitis mouse model, we investigated *Lactobacillus* *rhamnosus* strain LDTM 7511 originating from Korean infant feces as a putative probiotic strain for IBD. The strain LDTM 7511 not only alleviated the release of inflammatory mediators, but also induced the transition of gut microbiota from dysbiotic conditions, exhibiting the opposite pattern in the abundance of DSS colitis-associated bacterial taxa to the DSS group. Our findings suggest that the strain LDTM 7511 has the potential to be used as a probiotic treatment for IBD patients in comparison to *L. rhamnosus* GG (ATCC 53103), which has been frequently used for IBD studies.

## 1. Introduction

Inflammatory bowel disease (IBD) is an idiopathic chronic disease that involves inflammation of the gastrointestinal (GI) tract, including Crohn’s disease (CD) and ulcerative colitis (UC) [1]. Approximately 6.8 million cases of IBD were reported in 2017, and the incidence and prevalence of IBD are increasing worldwide [2]. Despite numerous studies and research, a definitive pathogenesis of IBD has not yet been elucidated. To date, scientific evidence has shown that IBD is driven by genetic defects and environmental factors [3], and the collapse of the mucosal barrier function could trigger the penetration of gut pathobionts into the mucosa, resulting in inflammation responses [4,5]. In addition, the role of gut microbiota in the pathogenesis of IBD has been focused on, and in particular, studies in germ-free mice have revealed that gut bacteria are essential for the initiation of intestinal inflammation [6]. However, whether the gut microbiota represent the cause or correlation of IBD has been a controversial topic [7,8]. In recent decades, microbiome-based therapeutics, such as prebiotics, probiotics, postbiotics, and fecal microbiota transplantation (FMT), have been proposed as promising strategies for IBD treatment [9].

In an inflamed gut, the mechanisms of action of probiotics have been found to inhibit pathogenic bacteria, improve epithelial and mucosal barrier function, modulate the immune response, and alter the gut microbiota composition [10,11]. The most commonly evaluated probiotic species or strains in IBD patients are *Escherichia coli* Nissle 1917 [12], *Lactobacillus* species [13], *Bifidobacterium* species [14], VSL#3 [15], and the yeast *Saccharomyces boulardii* [16]. On the basis of some clinical studies, orally administered *Lactobacillus rhamnosus* GG (LGG; ATCC53103), which is a commercial strain, has induced or maintained remission in UC [17] and CD patients [18,19]. Since the outcome of probiotics is not only host status-dependent [20], but also strain-specific [21], it is important to find a novel strain to be used to improve IBD symptoms.

Numerous experimental mouse colitis models have been developed that are similar to human UC or CD in terms of their etiology, pathology, and pathogenesis [22]. However, these models have not been developed with consideration of gut microbial dysbiosis. In particular, the gnotobiotic mice used in some studies are not sufficient for reflecting dysbiosis–host relationships due to their simplified gut microbiota [4]. The dextran sulfate sodium (DSS)-induced colitis model is widely used as one of the well-established murine colitis models. The administration of DSS in drinking water caused a loss of the epithelial barrier function and invasion of luminal contents into the lamina propria, resulting in the stimulation of immune responses [23]. According to Hernández-Chirlaque et al. [24], the absence of enteric bacteria in germ-free mice attenuated intestinal inflammation by DSS, suggesting that the gut microbiota is required for the development of DSS colitis. Even if DSS colitis does not reflect genetic defects linked to human IBD, this model not only has human UC-like pathogenesis and therapeutic responses, but also induces dysbiosis from an intact gut to reveal the role of gut microbiota in the development of IBD [25].

The objective of the present study is to explore the potential of a novel *L. rhamnosus* strain as probiotic candidate to be used for IBD treatment. In the present study, we selected *L. rhamnosus* strain LDTM 7511 originating from Korean infant feces based on in vitro potential probiotic characterization. The influence of the strain LDTM 7511 on intestinal inflammation and dysbiosis was investigated in a DSS-induced colitis murine model by comparing it with *L. rhamnosus* strain ATCC 53103.

## 2. Materials and Methods

### 2.1. Microbial Cultivation, Carbohydrate Utilization, and Enzymatic Activity

According to the ethical guidelines, feces from Korean infants under 3 years of age were obtained from 10 subjects. Briefly, the stool samples were collected in a sterilized stool container and immediately transferred to an anaerobic jar with a GasPak generator (Becton Dickinson, Sparks, MD, USA). Within 12 h, the samples were diluted and incubated in de Man Rogosa and Sharp (MRS) agar (Difco, Detroit, MI, USA) at 37 °C for 48 h in an anaerobic chamber (Coy Laboratory Products, Ann Arbor, MI, USA). Among the selected isolates based on the physiological characteristics of lactic acid bacteria (LAB), seven *Lactobacillus rhamnosus* strains were identified by 16s rRNA sequencing. Following the manufacturer’s instructions, API 50 CHL and API ZYM (BioMérieux, Marcy l’Etoile, France) were used to determine the microbial ability to ferment, oxidize, and assimilate 49 carbohydrates and enzyme activity to degrade 19 substrates. The *L. rhamnosus* ATCC 53103 strain was used in comparison for probiotic properties. The atmosphere for bacterial culture consisted of 5% CO_2_, 10% H_2_, and 85% N_2_ in an anaerobic chamber.

### 2.2. Antibiotic Resistance, Biogenic Amine Production, and Hemolytic Activity

The minimum inhibitory concentrations (MICs) were determined in LAB susceptibility medium (LSM) [26] using M.I.C.Evaluator Strips (Oxoid, Cambridge, UK). The microbiological breakpoints of antibiotics were in accordance with the guidelines proposed by the European Food Safety Authority [27], the National Committee for Clinical and Laboratory Standards Institute [28], and Danielsen and Wind [29]. The ability of the selected *L. rhamnosus* strains to produce amines (tyramine, cadaverine, putrescine, and histamine) was identified according to the protocol described by Bover-Cid and Holzapfel [30]. Hemolytic activity was found in blood agar supplemented with 5% (v/v) defibrinated sheep blood (Kisan Bio, Seoul, Korea). *Enterococcus faecalis* ATCC 29212 and *Staphylococcus aureus* ATCC 25923 were used as positive controls for biogenic amine formation and hemolysis, respectively.

### 2.3. Probiotic Properties

#### 2.3.1. Adhesion Ability

The human colorectal adenocarcinoma HT-29 cell line was grown on Roswell Park Memorial Institute 1640 (RPMI 1640, Gibco, Carlsbad, CA, USA) supplemented with 10% heat-inactivated fetal bovine serum, 100 units/mL penicillin, 100 μg/mL streptomycin, and 0.25 μg/mL amphotericin B. Prior to bacterial treatment, HT-29 was seeded in 12-well plates at 2.0 × 10^5^ cells/well and incubated in 5% CO_2_ atmosphere at 37 °C until a confluent monolayer formed. To promote nutrient adaptation, the tested LAB strains were subcultured at least two times in RPMI medium supplemented with 10% MRS medium. The bacterial overnight culture was harvested, washed, and diluted to a 1.0 optical density at 600 nm (approximately 3.0 × 10^8^ CFU/mL) using RPMI 1640 medium without antibiotics. A total of 1 mL of each LAB strain was inoculated on the confluent HT-29 cell layer. After incubation for 90 min, each well was washed twice with saline, and the cell layer was detached with trypsin-EDTA (Gibco, USA). The attached bacteria were enumerated on MRS agar using the drop plate method.

#### 2.3.2. Bacterial Survival in a GI Tract Model

The in vitro GI tract simulation model described in previous reports [31,32] was used, and the constituents and concentrations of chemicals were based on [32] (p. 10046). Briefly, 7 mL of the bacterial culture (5.0 × 10^8^ CFU/mL) was harvested, washed, and resuspended with 3 mL of synthetic saliva. After incubation for 5 min under aerobic conditions, 6 mL of gastric juice was added (final pH 3.0) and incubated for 90 min in an anaerobic atmosphere. Subsequently, 6 mL of duodenal juice, 3 mL of bile juice, and 1 mL of NaHCO_3_ were added and incubated for 2 h. Bacterial enumeration was performed during the initial and final steps on MRS agar using the drop plate method.

#### 2.3.3. Antibacterial Activity

Pathogenic indicators implicated in the development of IBD were selected based on previous clinical reports [33,34]: *Helicobacter pylori* ATCC 43504^T^, *Campylobacter coli* ATCC 33559^T^, and *Mycobacterium avium* subsp. *paratuberculosis* ATCC 19698^T^ (MAP). Cultures of *H. pylori* ATCC 43504^T^ and *C. coli* ATCC 33559^T^ were maintained on Columbia agar (Difco) supplemented with 7% defibrinated sheep blood at 37 °C for 72 h under microaerophilic conditions consisting of 7.5% CO_2_, 7.5% H_2_, 5% O_2_, and 80% N_2_. The MAP strain was grown in Middlebrook 7H9 broth base agar (Sigma-Aldrich, St. Louis, MO, USA) supplemented with 2 mL/L glycerol and 50 mL/L Middlebrook ADC growth supplement at 37 °C for 4 weeks. Colonies of pathogenic indicators were collected, suspended in saline, diluted to OD_600_ 1.0, and mixed with the supernatant of *L. rhamnosus* strains in the same volume. After 5 min incubation, bacterial adenosine triphosphate (ATP) was detected based on luminescence (RLU) using a BacTiter-Glo^TM^ Microbial Cell Viability Assay Kit (Promega, Madison, WI, USA) [35]. Sterilized fresh MRS medium was used as a negative control. Because of the cell wall and doubling time [36,37], MAP was co-incubated with LAB supernatant for 3 h before ATP detection.

#### 2.3.4. Inhibition of Nitric Oxide (NO) Production

The *Mus musculus* macrophage RAW264.7 cell line was grown in DMEM supplemented with 10% heat-inactivated fetal bovine serum, 100 units/mL penicillin, 100 μg/mL streptomycin, and 0.25 μg/mL amphotericin B (Gibco, Carlsbad, CA, USA). Cells were seeded in 24-well plates at 1.0 × 10^5^ cells/mL and stabilized for 2 h prior to the treatment of *E. coli* O111:B4 lipopolysaccharides (LPS; Sigma-Aldrich, St. Louis, MO, USA). Overnight bacterial cultures were harvested, washed, diluted to OD_600_ 1.0 with saline, and heated at 110 °C for 15 min. Complete bacterial death was confirmed before use. After the cell medium was withdrawn, 10% heat-killed bacteria and 90% LPS-supplemented DMEM medium were added (final LPS of 1 μg/mL). Saline with or without LPS-supplemented DMEM was used as a positive and negative control, respectively. After incubation for 48 h, the concentration of NO was determined by measuring the amount of nitrite in the cell culture supernatant by Griess reagent (Promega, Madison, WI, USA), according to the manufacturer’s instructions. Absorbance was measured at 540 nm.

### 2.4. In Vivo Experimental Design

The *L. rhamnosus* strains were lyophilized using trehalose (44.4%) as a cryoprotective agent and stored at –80 °C. Bacterial viability and contamination were confirmed once a week. Thirty-two female C57BL/6J mice of six-weeks-old were purchased from Daehan Bio Link Co., Ltd., Korea. Mice were kept in a controlled facility: temperature of 20–24 °C; humidity of 50%–55%; and 12 h light and 12 h dark cycle. The mice were fed with a standard laboratory chow diet and water *ad libitum*. After stabilization for 7 days, mice were orally provided daily with lyophilized LAB (10^9^ CFU/day) for 14 days, and acute colitis was then induced by the administration of 1.5% DSS (MW 36–50 kDa) in drinking water ad libitum for 6 days, followed by a recovery period of 6 days, and then mice were sacrificed. During the DSS colitis induction and recovery period, mice were orally administered LAB daily at 10^9^ CFU/day until sacrifice. Normal and DSS groups were only provided with 1× phosphate buffered saline (PBS) as the vehicles.

### 2.5. Intestinal Inflammation Biomarkers and Histology

The fecal sample was divided into three for triplicate repeats, and distilled water (DW) was added at a ratio of 50 mg/mL. Feces were completely suspended by vortexing and then centrifuged at 10,000× *g* for 3 min. The aliquots of the supernatants were stored at –80 °C. Mouse lipocalin–2/NGAL (Lcn–2) and myeloperoxidase (MPO) activities in fecal supernatants were quantified by commercially available ELISA kits (R&D Systems, Minneapolis, MN, USA), according to the manufacturer’s instructions. The amount of hemoglobin in feces was quantified by using the luminol reaction protocol suggested by Quickenden and Creamer [38], with some modifications. To generate a standard curve of hemoglobin, lyophilized human hemoglobin (Sigma-Aldrich, St. Louis, MO, USA) was reconstituted with DW at a concentration of 5 mg/mL, and then two-fold diluted serially. Fecal supernatant and standard samples were mixed with the luminol reagent at a ratio of 1:1, and the sole addition of DW was used for the background signal. Luminescence was immediately detected by the SpectraMax M4 Microplate/Cuvette Reader (Molecular Devices, San Jose, CA, USA).

At sacrifice, serum was isolated from mouse total blood and maintained at –80 °C. Serum C-reactive protein (CRP) was quantified using the Mouse C-Reactive Protein/CRP Quantikine ELISA Kit (R&D Systems, Minneapolis, MN, USA).

The distal colon samples were fixed in 10% buffered formalin phosphate (Sigma-Aldrich, St. Louis, MO, USA), and then paraffin-embedded and stained with hematoxylin and eosin (H&E) by the Contract Research Organization of the LOGONE Bio Convergence Research Foundation (Seoul, Korea). Histological score was determined using a previously published criteria [39] (p. 4562).

The total RNA was extracted following to the RNeasy Plus Mini Kit (Qiagen, Hilden, Germany) protocol. cDNA was synthesized using the PrimeScipt RT reagent Kit (Takara Korea Biomedical Inc., Seoul, Korea). Gene amplification was carried out according to the iQ SYBR Green Supermix (Bio-Rad Laboratories, Hercules, CA, USA) protocol. Data were normalized with the housekeeping β-actin expression level. The primers used are listed in Table S1.

### 2.6. Gut Microbiota Analysis

DNA was extracted from cecum samples according to the protocol of the QIAamp PowerFecal Pro DNA Kit (Qiagen, Hilden, Germany). The 16s rRNA gene library was constructed according to Illumina’s instructions [40]. The final PCR product was sequenced by Macrogen (Seoul, Korea) using a Miseq sequencer (Illumina Inc., San Diego, CA, USA). In Quantitative Insight into Microbial Ecology (QIIME 2), sequence reads were preprocessed, demultiplexing, trimming, and truncating low-quality regions. In addition, taxonomy assignment based on Greengenes reference and rooted/unrooted phylogenetic trees was conducted. Further data analysis and visualization were performed in the *R* phyloseq package [41] and GraphPad Prism (version 8.3.0; GraphPad software Inc., San Diego, CA, USA). Phylogenetic distances between groups were calculated from the generalized UniFrac (GUniFrac) [42]. The negative binomial distribution in differential abundance in comparison with the DSS group was determined using the DESeq2 package (BaseMean > 1 and adjusted *p* < 0.05) [43]. To find colitis-associated bacterial taxa, the genera belonging to the differential abundance in DESeq2 analysis were agglomerated at a genus level. Subsequently, Pearson correlation analysis of the bacterial taxa and inflammation biomarkers was carried out.

### 2.7. Statistical Anaysis

Statistical significance was analyzed in one-way analysis of variance (ANOVA) and student’s *T*-test to evaluate differences in discrete variables between the samples using GraphPad Prism. The ADONIS permutation-based statistical test was conducted in R. Pearson correlation analysis was conducted in a two-tailed test with a 95% confidence interval in GraphPad Prism.

### 2.8. Assession Numbers

The nucleotide sequences of 16s rRNA genes have been deposited in the GenBank database under the accession numbers MT318644–MT318650 for the seven *L. rhamnosus* strains isolated (strains LDTM 7511–7517). Metagenome 16s rRNA gene sequences have been deposited in the GenBank Sequence Read Archive (SRA) database as PRJNA623502.

### 2.9. Research Ethical Standards

The mothers of subjects gave their informed consent for inclusion on behalf of their infant, prior to participating in the study. Stool sample collection was conducted in accordance with the Declaration of Korea, and the protocol was approved by the Ethics Committee of Seoul National University (IRB Approval No. 1702/011-004). For the in vivo experiment, this study was carried out in accordance with the guidelines by the Korean Association for Laboratory Animals, and the protocol was approved by the Institutional Animal Care and Use Committee of Seoul National University (Approval No. SNU-170811-2-4).

## 3. Results

### 3.1. Metabolic Profiles and Safety Assessment of L. rhamnosus Strains

**Figure 1:**
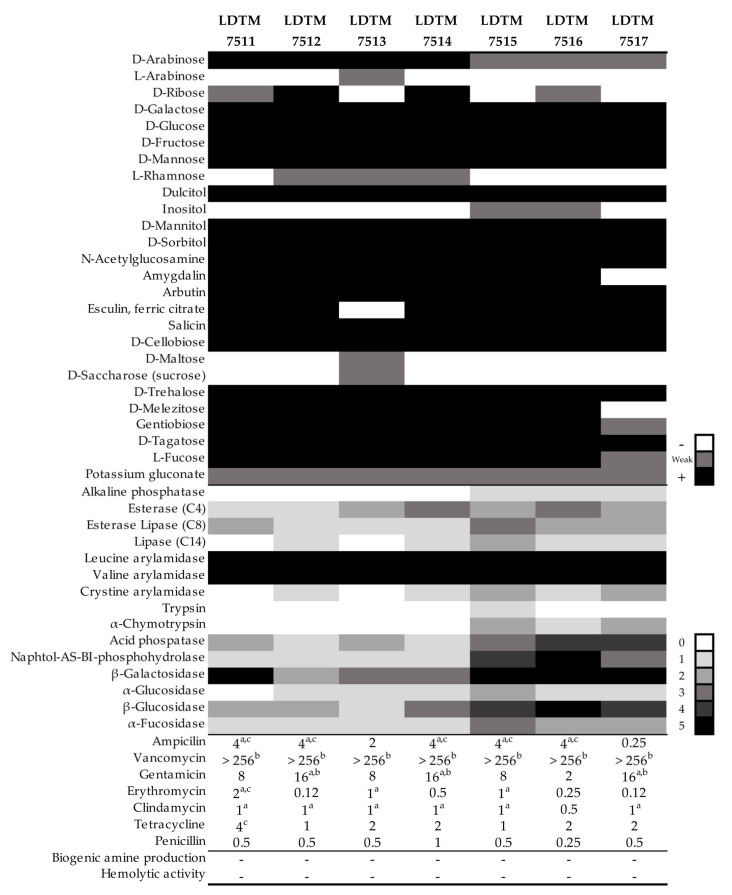
Fermentative/enzymatic profiles and safety assessment of isolated *Lactobacillus rhamnosus* strains. The legend on the right indicates the contrast intensity in each profile. No reaction in fermentative/enzymatic profiles was not indicated. Antibiotic resistance was determined based on the microbiological breakpoints suggested by EFSA (2012), CLSI (2010), and Danielsen and Wind (2003), and the minimum inhibitory concentration (MIC) values (μg/mL) above each criterion are indicated by superscripts of a, b, and c, respectively.

The *L. rhamnosus* strains isolated from Korean infant feces showed the selective utilization of carbohydrates and substrates (Figure 1). The strain LDTM 7511 did not ferment L-arabinose, L-rhamnose, inositol, D-maltose, and D-sucrose, and showed potent leucine and valine arylamidase and β-galactosidase activity. All strains displayed resistance to vancomycin in excess of 256 μg/mL of MICs, but this is not a mandatory criterion because *L. rhamnosus* species is inherently resistant to vancomycin [27]. All tested strains did not exhibit biogenic amine production and hemolysis activity.

### 3.2. Probiotic Properties of L. rhamnosus LDTM 7511

Among the isolates, the strain LDTM 7511 had more prominent probiotic properties than others. The LPS-stimulated RAW264.7 cells treated with supernatants of LDTM 7511 and ATCC 53103 produced 7.2 ± 0.03 and 11.5 ± 0.06 μM of nitric oxide, respectively (Table 1). The adherence to HT-29 cells was similar for the two strains. In the in vitro human GI tract model, the survival rate of strain LDTM 7511 was lower than that of ATCC 53103, but there was no significant difference. The supernatants of the two *L. rhamnosus* strains significantly inhibited the viability of IBD-related pathogenic indicators compared to the negative control.

### 3.3. Anti-Inflammatory Effect of Strain LDTM 7511 in DSS-Induced Colitis Mice

**Figure 2:**
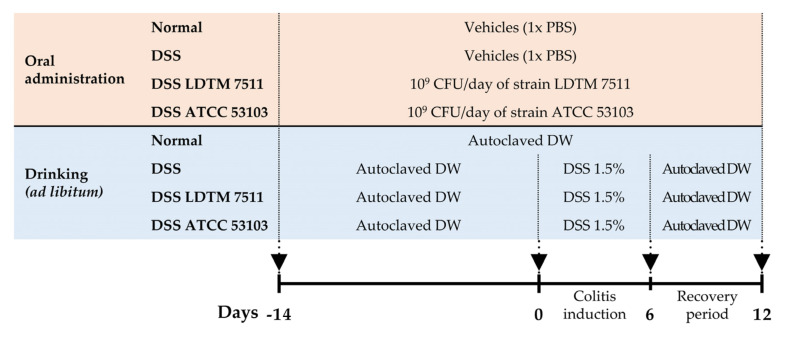
Overview of the in vivo experiment. Based on LAB and DSS treatment, the experimental group consisted of Normal, DSS, DSS LDTM 7511 and DSS ATCC 53103.

The in vivo experimental overview is shown in Figure 2. Normal refers to a group without DSS treatment, and DSS indicates a colitis-induced group by DSS treatment. Both groups received the vehicle instead of LAB strains.

The DSS administered with the DSS LDTM 7511 group had a significantly longer colon length (adjusted *p* = 0.0044) and smaller spleen weight (adjusted *p* = 0.0254) than the DSS group, which was similar to the normal group (Table 2). However, in all experimental groups, no considerable loss of body weight was observed on the day of sacrifice compared to the initial date of DSS treatment. In the DSS group, Lcn–2 levels increased during the recovery period, even after DSS treatment was discontinued. On the other hand, the MPO level was maintained until the recovery period. The primary and secondary neutrophil-related molecules, Lcn–2 and MPO, were significantly lower in mice provided with strain LDTM 7511 compared to the DSS group, before and after the recovery period of 6 days (adjusted *p* < 0.0001, except *p* = 0.0002 in MPO after DSS injury). *L. rhamnosus* ATCC 53103 also attenuated the production of fecal markers compared to the DSS group, but its impact was lower than that of the DSS LDTM 7511 group. In LAB administered groups, the amount of hemoglobin in feces was less than that of the DSS group, with no statistical significance, and hemoglobin was not detected in all groups after the recovery phase. The level of CRP, also known as pentraxin 1, similar to the normal group, was observed in the serum of LDTM 7511-administered mice (adjusted *p* = 0.0021, compared to the DSS group).

Due to the variation between individuals, no statistical significance was observed in the mRNA expression of pro-inflammatory cytokines. However, the DSS LDTM 7511 group showed downregulated expression levels in the determined cytokines compared to the DSS group. In the case of the DSS ATCC 53103 group, IFN–γ, IL–1β (adjusted *p* = 0.0285), and IL–6 were upregulated.

The expression levels of genes encoding adherens and tight junction proteins between intestinal epithelial cells were not different between DSS LDTM 7511 and DSS groups, except for claudin-2. The expression of claudin-2, which is known to be a mediator of leaky gut in an inflamed gut [44], was downregulated in the LAB-treated groups, and the DSS ATCC 53103 group revealed 0.38-fold expression for the DSS group (adjusted *p* = 0.0014).

**Figure 3:**
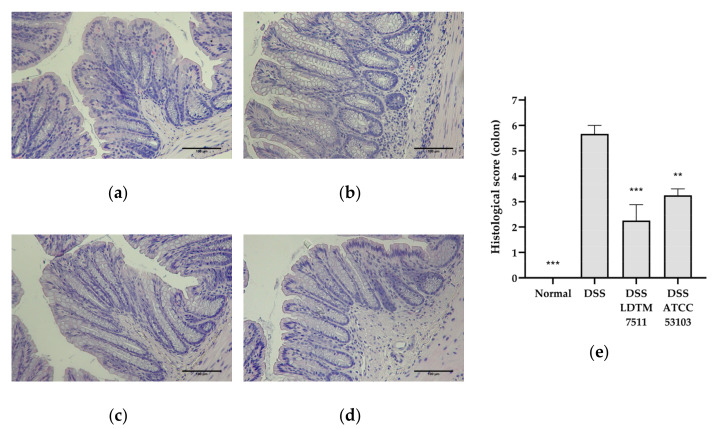
Representative colonic architecture and histological score in hematoxylin and eosin (H&E) staining. (**a**) Normal group; (**b**) DSS group; (**c**) DSS LDTM 7511 group; (**d**) DSS ATCC 53103 group; (**e**) Histological score (mean ± SEM, n = 4 in each group). Original magnification, 20×. Scale bars, 100 μm. In (**e**), significance is indicated as follows: ** *p* < 0.01 and *** *p* < 0.001, compared to the DSS group.

In the histological analysis, the DSS group showed mucosal leukocyte infiltration and irregular crypts (Figure 3). On the other hand, the distal colon in LAB-administered groups revealed partial infiltrates in the submucosa and partial irregular crypt architectures. The DSS LDTM 7511 group demonstrated a relatively intact colonic architecture compared to the DSS ATCC 53103 group, but the degree of leukocyte infiltration was similar between the LAB-treated groups.

### 3.4. Gut Microbiota Modulation Effect of Strain LDTM 7511 in DSS-Induced Colitis Mice

**Figure 4:**
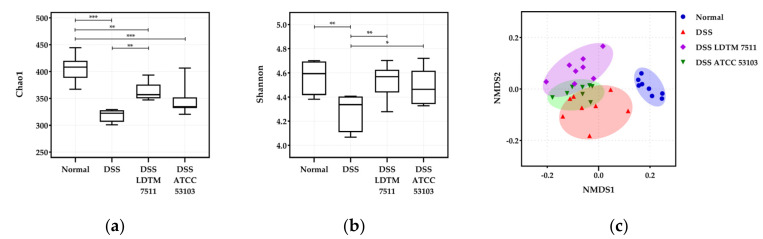
Alpha and beta diversity. (**a**) Chao1; (**b**) Shannon; (**c**) non-metric multidimensional scaling (NMDS) plot of GUniFrac distances. Statistical significance is indicated as follows: * *p* < 0.05, ** *p* < 0.01, and *** *p* < 0.001; n = 8 in each group.

The alpha diversity was estimated by Chao1 (F = 18.49, *p* < 0.0001) and Shannon indices (F = 4.742, *p* = 0.0102) (Figure 4a,b). Although the bacterial richness decreased in DSS colitis-induced groups, the Chao1 index in the DSS LDTM 7511 group significantly increased compared to the DSS group (adjusted *p* = 0.0019). Shannon index values in LAB-treated groups were not only similar to the normal group, but also significantly higher than the DSS group (adjusted *p* = 0.0091 in DSS LDTM 7511; 0.0389 in DSS ATCC 53103). The ordination of the phylogenetic multivariate distance between groups was expressed as a non-metric multidimensional scaling (NMDS) plot (Figure 4c). DSS colitis groups were separated from the normal group, and separation of the DSS LDTM 7511 cluster from the DSS group was found among the DSS-treated groups. The ADONIS permutation-based statistical analysis with 999 permutation was determined (R^2^ = 0.4594 and *p* = 0.001).

**Figure 5:**
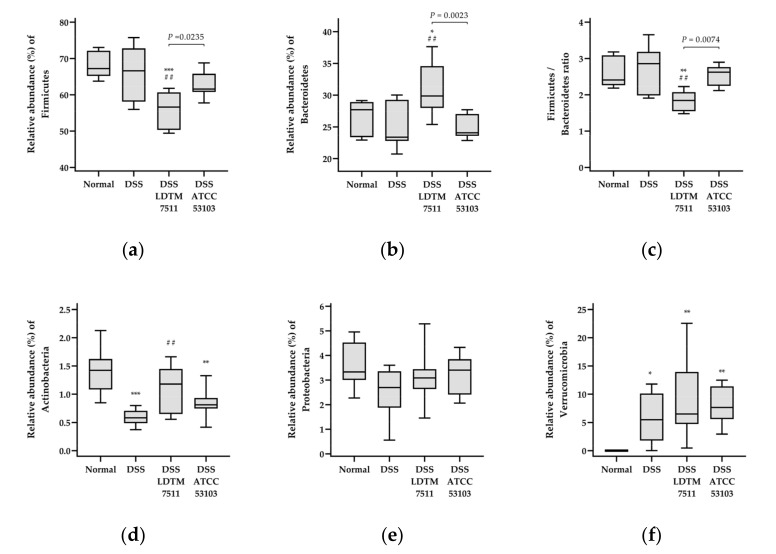
Relative abundance (%) of the dominant phylum and Firmicutes/Bacteroidetes ratio. (**a**) Firmicutes; (**b**) Bacteroidetes; (**c**) Firmicutes/Bacteroidetes (F/B) ratio; (**d**) Actinobacteria; (**e**) Proteobacteria; (**f**) Verrucomicrobia. Statistical significance is indicated as follows: * *p* < 0.05, ** *p* < 0.01, and *** *p* < 0.001, compared to the normal group; ## *p* < 0.01, compared to the DSS group; n = 8 in each group.

Relative abundances of the dominant phylum in the mouse gut are indicated in Figure 5 and Supplementary Data S1. Notably, the populations in mice that were administrated strain LDTM 7511 showed a different pattern from other groups. There were no significant differences in the relative abundance of Firmicutes and Bacteroidetes between normal and DSS groups, but the DSS LDTM 7511 group was significantly different compared to the non-LAB-treated groups (Figure 5a,b). The ratio of Firmicutes/Bacteroidetes (F/B) in the DSS LDTM 7511 group was considerably reduced (compared to the normal group, *p* = 0.0032 and DSS group, *p* = 0.0011) (Figure 5c). Moreover, the abundances in the two major phyla were also significantly different from the DSS ATCC 53103 group. The population of Actinobacteria was reduced in the DSS and DSS ATCC 53103 groups, but the DSS LDTM 7511 group was no different in terms of the Actinobacteria compared to the normal group (Figure 5d). In all DSS colitis-induced groups, Verrucomicrobia populations increased (Figure 5f). There were no considerable differences between groups in the Proteobacteria (Figure 5e).

**Figure 6:**
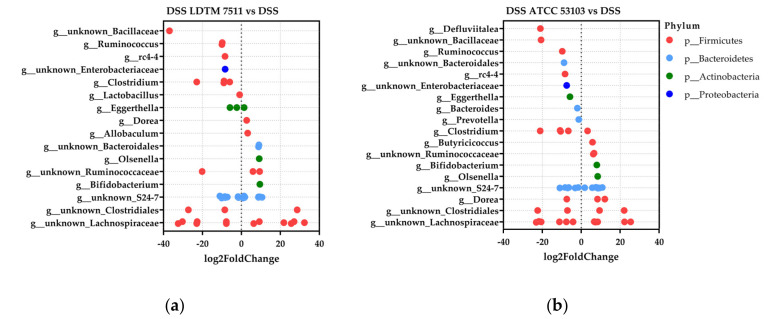
Differential abundance determined using the DESeq2 package. Compared to the DSS group, log2 fold-change in (**a**) DSS LDTM 7511 and (**b**) DSS ATCC 53103 groups. Criteria for inclusion: mean of normalized counts for all samples (BaseMean) > 1 and Benjamini–Hochberg adjusted *p* value (padj) < 0.05; *n* = 8 in each group.

The negative binomial distribution in differential abundance between the DSS group and each LAB-treated group was determined in DESeq2 (Figure 6 and Supplementary Data S2 and S3). *Bifidobacterium* and *Olsenella*, which belong to the Actinobacteria phylum, commonly increased in LAB-administered groups, compared to the DSS group. On the other hand, fold-change (log2) values below 0 were commonly observed in *Ruminococcus*, *rc4-4*, uncultured Bacillaceae, and Enterobacteriaceae in each LAB-treated group in comparison to the DSS group.

**Figure 7:**
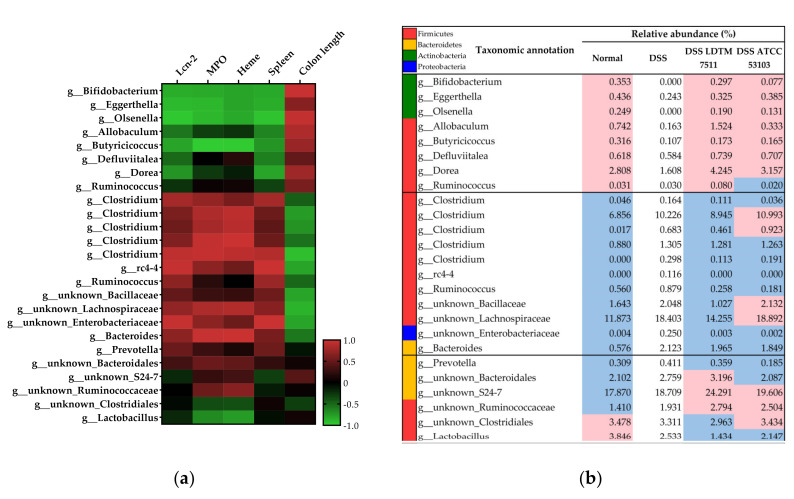
DSS colitis-associated bacterial taxa and their relative abundances (%). (**a**) Pearson correlation between inflammation biomarkers and bacterial taxa at a genus level (n = 32). (**b**) Relative abundance in experimental groups (*n* = 8 in each group). The population values higher and lower than the DSS group are shown in red and blue, respectively.

The DESeq2 data were agglomerated at the genus level to find the correlation between the genus and inflammatory biomarkers (Supplementary Data S4 and S5). Among the tested colitis markers, three fecal biomarkers and the spleen weight were selected as indicators with a positive correlation with inflammation, while colon length was selected as an indicator with a negative correlation in DSS colitis [45,46] (see Table 2). The genera belonging to Actinobacteria (*Bifidobacterium*, *Eggerthella*, and *Olsenella*), *Allobaculum*, *Butyricoccus*, *Defluviitalea*, *Dorea*, and *Ruminococcus* (assigned species as *Ruminococcus*) showed a negative correlation with the colitis severity, displaying a positive correlation with the colon length (Figure 7a). On the contrary, *Clostridium*, *rc4-4, Ruminococcus* (assigned species as *Ruminococcus lactaris*)*, Bacteroides*, uncultured Bacillaceae, Lachnospiraceae, and Enterobacteriaceae exhibited a positive correlation with the inflammation severity, showing a negative correlation with the colon length. Therefore, we divided the genera into two groups associated with gut inflammation, excluding *Prevotella*, *Lactobacillus*, uncultured Bacteroidales, S24-7, Rumimococcaceae, and Clostridiales (Figure 7a). Notably, in the LDTM 7511-administered group, the pattern of increasing and decreasing abundance was similar to the normal group (Figure 7b). In addition, the genus associated with severe inflammation had a lower abundance in the DSS LDTM 7511 group than that of the DSS group. In contrast, the abundances related to the low inflammatory state were higher than that of the DSS group.

## 4. Discussion

Probiotics have therapeutic effects that alleviate inflammation in patients with IBD [47]. Findings from animal colitis models have shown that probiotics modulate the immune response, gut microbiota composition, and GI environmental conditions [48]. According to the results of Kekkonen et al. [21], the anti-inflammatory effect of probiotics was shown to be strain-specific in healthy adults. In addition, Zhai et al. [49] reported that the administration of two strains of the next-generation probiotic *Akkermansia muciniphila* displayed strain-specific characteristics in alleviating DSS chronic colitis, which were attributed to genetic differences between the two strains. *L. rhamnosus* ATCC 53103 (LGG) is a commensal bacterium isolated from a healthy human GI tract in 1983 [50]. To the best of our knowledge, *L. rhamnosus* ATCC 53103 is the only strain of *L. rhamnosus* species that has been used thus far in IBD clinical studies. The efficacy of ATCC 53103 supplementation is still controversial [51], but some clinical observations support its potential to be a therapeutic agent for preventing relapse in IBD patients [17,18,19]. In the current study, we evaluated the effect of *L. rhamnosus* strain LDTM 7511 on the attenuation of inflammation and normalization of intestinal microflora in DSS colitis by comparing it to the strain ATCC 53103. 

Regarding the safety issues of probiotic ingestion, the strain LDTM 7511 displayed no β-glucuronidase activity known to be a potential carcinogenic agent in the intestine [52,53]. On the other hand, relatively weak β-glucosidase activity was observed in the strain LDTM 7511 (Figure 1). According to a clinical report described by Mroczyńska et al. [53], there was no significant difference in the stool β-glucosidase activity between healthy adults and IBD patients, indicating that β-glucosidase is a less valuable indicator of IBD than β-glucuronidase. The strain LDTM 7511 exhibited strong aminopeptidase activity, including valine and leucine arylamidase, as well as β-galactosidase activity, which is responsible for the hydrolysis of lactose. In addition, LDTM 7511 did not ferment L-arabinose and L-rhamnose, which are unusual sugar forms in nature (Figure 1).

Clinical evidence in recent decades has shown the role of certain pathogens in the etiopathogenesis of IBD. The most indispensable pathogens are MAP, *Campylobacter*, adherent-invasive *E. coli*, and *Helicobacter* species [33,34]. MAP has been considered an etiological factor for the development of CD due to TNF–α induction caused by invading intestinal epithelial cells and eliciting a dysfunction of phagocytes [54]. *Campylobacter* is a human intestinal pathogen that causes diarrhea, abdominal pain, fever, and sometimes gut bleeding. *Campylobacter jejuni*, *Campylobacter concisus*, and *Campylobacter hominis* have been frequently reported in relation to chronic intestinal disease. In particular, *C. coli* infection is one of the causes of bacterial enteritis in developed countries, and its ability to invade and transverse the intestinal epithelium has been reported [55,56]. *Helicobacter pylori* is a well-known species that causes several gastrointestinal diseases. Interestingly, recent studies have elucidated the protective role of *H. pylori* in an inflamed gut by inducing the expression of Foxp3+ Tregs and suppressing inflammatory cytokines [57]. However, the trade-off between a protective effect in IBD and risk in gastric disease by *H. pylori* infection is not completely understood [58]. In this study, the supernatants of tested *L. rhamnosus* strains inhibited the viability of the tested pathogenic indicators (Table 1). Nitric oxide released by phagocytes has been associated with the initiation and maintenance of inflammation in IBD [59]. Although the two *L. rhamnosus* strains tested showed similar abilities of adhesion and survival in in vitro GI adaptation, the strain LDTM 7511 substantially inhibited the production of NO in LPS-stimulated RAW264.7 macrophages in comparison to strain ATCC 53103 (Table 1).

Dextran sulfate sodium has a highly negative charge in the sulfate group, causing the erosion of intestinal epithelial cells, which increases the penetration of immune cells and release of pro-inflammatory cytokines [45]. The DSS-induced animal colitis model has the advantage that it can easily induce colitis similar to ulcerative colitis. The reproducibility of this model depends on various factors, such as the mouse strain, facility conditions, molecular weight and source of DSS, concentration, and duration [60]. The DSS colitis model can be divided into acute and chronic models, depending on the frequency of recovery and duration of DSS exposure. The range of DSS dosage commonly varies from 1% to 5%. In this study, we induced low-grade inflammation with DSS 1.5%, because the oral administration of live bacteria in a severely inflamed gut with collapse of the mucus barrier might cause side effects, such as bacteremia [61,62]. Furthermore, it was confirmed that there was no undesirable effect of the tested *L. rhamnosus* strains when administered for 4 weeks from initial colonization to sacrifice (Figure 2). Weight loss has often been considered as a biomarker of inflammation in DSS colitis, but it is not suitable as a biomarker in low-grade inflammation. As per previous reports [63], the mild colitis induced by DSS 1.5% did not cause significant body weight reductions (Table 2). As described in Section 3.3 (Table 2), the strain LDTM 7511 not only significantly attenuated inflammation by reducing neutrophil-related proteins, but also improved the physiological features. Moreover, the mice that received LDTM 7511 showed a considerably lower expression of pro-inflammatory cytokine genes than the DSS group.

Several studies have reported that a major pathological feature of IBD is an increased intestinal permeability due to the loss of adherens and tight junctions between intestinal epithelial cells. In healthy conditions, the cadherins belonging to adherens junctions dimerize with cadherins on adjacent cells, and tight junction proteins, such as Zonula occludens-1 (ZO-1), occludin, and claudin families, in the upper side of the adherens junction, enhance the cell-to-cell binding. In the inflamed intestine, the expression of these junction proteins was generally downregulated, except for claudin-2 [64]. Claudin-2 is mainly observed in the apical region of colonocytes, and this protein is considered to increase the permeability by forming water and a small cation channel [44]. The upregulation of claudin-2 has been observed in an IBD and colitis model [65,66]. In terms of our in vivo results, the gene expression of adherens and tight junction proteins between normal and DSS groups supported the previous results (Table 2). However, the expression of claudin-2 was significantly downregulated in the DSS ATCC 53103 group, and mice provided with the strain LDTM 7511 did not display a significant differential expression in junction-related genes in comparison to the DSS group. These results suggest that the underlying probiotic mechanisms might be strain-specific.

Emerging evidence indicates that gut microbiota are implicated in the development of IBD. However, a clear definition of dysbiosis and homeostasis in intestinal microflora has not been established. Efforts have been made to find differential microbial groups, which can be used as an indicator of dysbiosis, between IBD patients and healthy humans using clinical metadata analysis. Even though the pattern of microbial alteration has been reported differently by host genetics, disease type, status, and individuals, bacterial taxa which are commonly altered in IBD patients have been identified as Clostridiales, Bifidobacteriaceae, *Faecalibacterium prausnitzii*, *Bacteroides*, Enterobacteriaceae, and Verrucomicrobiaceae [67,68,69,70]. In the DSS colitis murine model, *Bacteroides distasonis*, *Clostridium ramosum*, *Akkermansia muciniphila*, and Enterobacteriaceae have been reported as microorganisms associated with DSS colitis [71]. However, more information is still needed to define dysbiosis in IBD or DSS colitis. In the present study, we defined the microbiome in the DSS group as having a dysbiosis status and compared it with other groups. Furthermore, we identified microbial taxa associated with inflammatory biomarkers (Figure 7a), and the results support the general consensus on the key role of the gut microbiota in the pathogenesis of IBD. In particular, it is noteworthy that the group DSS LDTM 7511 exhibited an increase and decrease pattern of the selected genera similar to the normal group (Figure 7b). A number of DSS colitis studies have reported a significant reduction in *Bifidobacterium* and induction in Enterobacteriaceae and *Bacteroides* [72,73,74]. *Akkermansia muciniphila* belonging to the phylum Verrucomicrobia is one of the generally increasing species in DSS colitis [75], and its increased abundance in DSS-treated groups was observed in this study (Figure 5f). Current theories on the mechanisms of inflammation-driven dysbiosis propose that an increased lumen oxygen level from leaky gut induces an overgrowth of facultative anaerobes, such as Enterobacteriaceae, and strictly inhibits anaerobes, including *Bifidobacterium* species. In addition, Enterobacteriaceae utilize carbohydrate and sialic acids released from the mucus barrier by mucus-degrading bacteria, such as *Bacteroides vulgatus* and *Akkermansia muciniphila*, resulting in an increased invasion of lumen contents into the mucosa and submucosa [76]. Our results support the current discoveries and highlight that the strain LDTM 7511 might normalize or beneficially modulate gut microbiota from DSS-driven dysbiosis.

In conclusion, our results show that the *Lactobacillus rhamnosus* strain LDTM 7511 is a promising probiotic candidate for IBD treatment because the strain may alleviate inflammation and normalize bacterial dysbiosis in an inflamed gut. In the future, mechanism-based interactions between the host and gut microbiota and human trials will be needed to confirm the long-term safety and efficacy of strain LDTM 7511 consumption. Our findings highlight that the novel strain *L. rhamnosus* LDTM 7511 has the potential to assist in the treatment of IBD, and characterization of the probiotic properties at a strain level will offer the opportunity to find novel strategies for microbiome therapeutics.

## Figures and Tables

**Table 1:** In vitro characteristics of potential probiotics.

Probiotic Properties		LDTM 7511	ATCC 53103
Adherence to HT-29 (%)		3.766 ± 0.067	3.822 ± 0.126
Survival (Log, %) in in vitro GI tract		61.90 ± 0.008	64.46 ± 0.009
Inhibition on LPS-induced NO production (%) ^1^		54.17 ± 0.002 ***^,^^#^	27.03 ± 0.004 ***^,^^#^
Pathogenic bacteria inhibition ^2^	*H. pylori* ATCC 43504^T^	0.574 ± 0.052 *	0.589 ± 0.073 *
	*C. coli* ATCC 33559^T^	0.251 ± 0.091 **	0.325 ± 0.028 **
	MAP ATCC 19698^T^	0.267 ± 0.003 **	0.311 ± 0.084 *

Data is expressed as the mean ± standard deviation. ^1^ Fold inhibition (%) on positive control. Significance is indicated as follows: *** *p* < 0.001, compared to the positive control; # *p* < 0.001, significance between two *L. rhamnosus* strains. ^2^ Fold change in adenosine triphosphate (ATP) bioluminescence (RLUs) of *Helicobacter pylori* and *Campylobacter coli* strains to the negative control after 5 min of incubation. Fold change in ATP bioluminescence (RLUs) of the *Mycobacterium avium* subsp. *paratuberculosis* ATCC 19698^T^ (MAP) strain after 3 h of incubation in terms of the initial values. Significance is indicated as follows: * *p* < 0.05 and ** *p* < 0.01, compared to the negative control. The survival in the in vitro GI tract was analyzed by student’s *T*-test, and one-way analysis of variance (ANOVA) was applied to evaluate differences in discrete variables between the groups in the other results.

**Table 2:** Effect of *L. rhamnosus* strains on an inflamed gut in a dextran sulfate sodium (DSS)-induced colitis murine model.

Inflammation Biomarkers		Experimental Groups			
		Normal	DSS	DSS LDTM 7511	DSS ATCC 53103
Loss of body weight (%)		1.67 ± 0.87	2.79 ± 1.18	1.85 ± 1.14	2.83 ± 0.76
Colon length (mm)		83.02 ± 1.04 **	74.98 ± 2.06	83.17 ± 1.83 **	78.19 ± 1.50
Spleen (g)		0.075 ± 0.003 *	0.106 ± 0.008	0.077 ± 0.006 *	0.081 ± 0.011
Hemoglobin/feces (mg/g) ^1^		0.00 ± 0.00 ***	87.34 ± 14.65	65.54 ± 11.42	73.71 ± 8.87
Lcn–2/feces (ng/g)	After DSS Injury	20.39 ± 0.26 ***	365.07 ± 2.45	292.59 ± 0.75 ***	333.73 ± 8.81 **
	After recovery	15.31 ± 0.28 ***	2295.3 ± 18.84	482.57 ± 7.00 ***	675.81 ± 41.90 ***
MPO/feces (ng/g)	After DSS Injury	0.000 ± 0.00 ***	8.290 ± 0.42	6.046 ± 0.04 ***	7.531 ± 0.09
	After recovery	0.000 ± 0.00 ***	9.943 ± 0.56	6.368 ± 0.02 ***	6.600 ± 0.06 ***
Serum CRP (μg/mL)		3.721 ± 0.18 *	4.769 ± 0.31	3.283 ± 0.11 **	3.919 ± 0.34
Relative fold expression for DSS group	TNF–α	0.179 ± 0.02 *	1.000 ± 0.31	0.786 ± 0.18	0.265 ± 0.06 *
	IFN–γ	0.112 ± 0.01	1.000 ± 0.43	0.602 ± 0.10	1.717 ± 0.83
	IL–1β	0.202 ± 0.03	1.000 ± 0.22	0.914 ± 0.16	14.143 ± 6.61 *
	TGF–β	0.260 ± 0.04	1.000 ± 0.48	0.390 ± 0.07	0.213 ± 0.02
	IL–6	0.123 ± 0.02	1.000 ± 0.45	0.287 ± 0.06	2.365 ± 0.91
	Occludin	1.400 ± 0.03 **	1.000 ± 0.10	0.952 ± 0.07	0.737 ± 0.07 *
	ZO–1	1.364 ± 0.11 *	1.000 ± 0.10	0.986 ± 0.06	1.045 ± 0.08
	Claudin–2	0.663 ± 0.09	1.000 ± 0.16	0.659 ± 0.12	0.379 ± 0.03 **
	E–cadherin	1.270 ± 0.10 *	1.000 ± 0.04	0.951 ± 0.06	0.826 ± 0.03

Data is expressed as the mean ± standard error or the mean (SEM). ^1^ Amount of hemoglobin in feces was quantified before the recovery period. One-way analysis of variance (ANOVA) was applied to evaluate differences in discrete variables between the groups. Significance is indicated as follows: * *p* < 0.5, ** *p* < 0.01, and *** *p* < 0.001, compared to the DSS group. A significant positive or negative response is indicated by blue and red colors, respectively.

## Supplementary Materials

The following are available online at https://www.mdpi.com/2076-2607/8/6/845/s1: Table S1: List of primers used in this study; Supplementary Data S1: Relative abundance (%) in individual samples; Supplementary Data S2: Differential abundance of DSS LDTM 7511 over the DSS group in DESeq2; Supplementary Data S3: Differential abundance of DSS ATCC 53103 over the DSS group in DESeq2; Supplementary Data S4: Relative abundance (%) of the agglomerated group at a genus level; Supplementary Data S5: Pearson correlation between specific taxa and the inflammatory biomarker.
