# Supplementary material for: Anti-Inflammatory and Gut Microbiota Modulatory Effect of Lactobacillus rhamnosus Strain LDTM 7511 in a Dextran Sulfate Sodium-Induced Colitis Murine Model
Source: Microorganisms. 2020 Jun 4;8(6):845. doi: 10.3390/microorganisms8060845 (PMC7356973; doi:10.3390/microorganisms8060845)
Supplement: Supplementary file 1 [file microorganisms-08-00845-s001.zip › Supplementary files_rev/Microorganisms (2020) Supplementary Materials.pdf]

microorganisms

Table S1. List of primers used in this study.

Target	Forward sequence (5′–3′)	Reverse sequence (3′–5′)	References
TNF–α	ATGAGCACAGAAAGCATGA	AGTAGACAGAAGAGCGTGGT	[1]
IFN–γ	TTCTTCAGCAACAGCAAGGC	TCAGCAGCGACTCCTTTTCC	
TGF–β	CCTGCAAGACCATCGACATG	TGTTGTACAAAGCGAGCACC	
Occludin	CCTCCAATGGCAAAGTGAAT	CTCCCCACCTGTCGTGTAGT	
ZO–1	CCACCTCTGTCCAGCTCTTC	CACCGGAGTGATGGTTTTCT	
Claudin–2	TATGTTGGTGCCAGCATTGT	TCATGCCCACCACAGAGATA	
IL–1β	CCTTCCAGGATGAGGACATGA	TGAGTCACAGAGGATGGGCTC	[2]
IL–6	AGTTGCCTTCTTGGGACTGA	CAGAATTGCCATTGCACAAC	[3]
E–cadherin	ACTGTGAAGGGACGGTCAAC	GGAGCAGCAGGATCAGAATC	[4]
B-actin	TCC ATC ATG AAG TGT GAC GT	GAG CAA TGA TCT TGA TCT TCA T	[5]

1. Liu, T.; Shi, Y.; Du, J.; Ge, X.; Teng, X.; Liu, L.; Wang, E.; Zhao, Q. Vitamin D treatment attenuates 2,4,6-trinitrobenzene sulphonic acid (TNBS)-induced colitis but not oxazolone-induced colitis. *Sci. Rep.* **2016** 6, 32889. doi: 10.1038/srep32889.
2. Aoki, R.; Aoki-Yoshida, A.; Suzuki, C.; Takayama, Y. Protective effect of indole-3-pyruvate against ultraviolet b-induced damage to cultured HaCaT keratinocytes and the skin of hairless mice. *PLoS One* **2014** 9, e96804. doi: 10.1371/journal.pone.0096804.
3. Jeengar, M.K.; Thummuri, D.; Magnusson, M.; Naidu, V.G.M.; Uppugunduri, S. Uridine ameliorates dextran sulfate sodium (DSS)-Induced Colitis in Mice. *Sci. Rep.* **2017** 7, 3924. doi: 10.1038/s41598-017-04041-9.
4. Yang, X.; Sarvestani, S.K.; Moeinzadeh, S.; He, X.; Jabbari, E. Effect of CD44 binding peptide conjugated to an engineered inert matrix on maintenance of breast cancer stem cells and tumorsphere formation. *PLoS One* **2013** 8, e59147. doi: 10.1371/journal.pone.0059147.
5. Yin, R.; Tian, F.; Frankenberger, B.; de Angelis, M.H.; Stoeger, T. Selection and evaluation of stable housekeeping genes for gene expression normalization in carbon nanoparticle-induced acute pulmonary inflammation in mice. *Biochem. Biophys. Res. Commun.* **2010** 399, 531–536. doi: 10.1016/j.bbrc.2010.07.104.
